# The Rumen Microbiome Composition of Raramuri Criollo and European Cattle in an Extensive System

**DOI:** 10.3390/microorganisms12112203

**Published:** 2024-10-31

**Authors:** Adrian Maynez-Perez, Francisco J. Jahuey-Martínez, José A. Martínez-Quintana, Michael E. Hume, Robin C. Anderson, Agustín Corral-Luna, Felipe A. Rodríguez-Almeida, Yamicela Castillo-Castillo, Monserrath Felix-Portillo

**Affiliations:** 1 Facultad de Zootecnia y Ecología, Universidad Autónoma de Chihuahua, Chihuahua 31453, Chih., Mexico; amaynez@uach.mx (A.M.-P.); fjahuey@uach.mx (F.J.J.-M.); jomartinez@uach.mx (J.A.M.-Q.); acorral@uach.mx (A.C.-L.); frodrigu@uach.mx (F.A.R.-A.); ycastillo@uach.mx (Y.C.-C.); 2 Food and Feed Safety Research Unit, Southern Plains Area Research Center, United States Department of Agriculture, Agricultural Research Service, College Station, TX 77845, USA; mehume@suddenlink.net (M.E.H.); robin.anderson@usda.gov (R.C.A.)

**Keywords:** rumen microbiome, adapted breeds, heritage cattle

## Abstract

Understanding the relationship between Raramuri Criollo cattle (RC) and their microbial ruminal ecosystem will help identify advantageous characteristics of adapted cattle as alternatives to achieve sustainable beef production systems. Our objective was to characterize the rumen microbiome of RC in comparison to Angus and Hereford breeds (European, E) and the cross between them (E × RC). Ruminal fluid was collected from 63 cows in their second productive cycle after grazing in the same paddock for 45 d, in the dry (n = 28) and rain (n = 35) seasons. DNA from ruminal fluid was isolated for 16s rRNA gene next-generation sequencing. The data were analyzed with QIIME2 and compared against the SILVA 16s rRNA database. Beta diversity was different (*p* < 0.05) between RC and E in both seasons. A microbial core was represented by the most abundant phyla. Planctomycetes and Spirochaetes represented above 1% in the rain season and below 1% in the dry one, whereas Euryarchaeota was below 1% and around 3%, respectively. LEfSe analysis identified differentiated (*p* < 0.05) key microbial groups that explain the differences between lineages at different taxonomic levels, reflecting the ability of the rumen ecosystem of RC cattle to adapt to hostile environmental conditions by having microbial groups specialized in the degradation of highly fibrous content.

## 1. Introduction

Throughout the last decades, the conservation of genetic diversity from locally adapted livestock breeds has gained importance for animal production systems [1] as an alternative to mitigate climate challenges. These genetically adapted breeds contribute to food security in developing countries. In addition, their ability to survive in hostile environmental conditions makes them an important component of local biodiversity and a resource of particular traits that may contribute to reaching management goals for sustainability [2,3].

Raramuri Criollo cattle (RC) have been under natural selection for the last five centuries in northern Mexico, handled by local indigenous communities without the influence of crossbreeding with other exotic breeds [4]. The rusticity of RC has been the focus of different research groups in the USA and Mexico for the last 20 years. Currently, scientific data have revealed desirable traits of landscape distribution, heat tolerance, and diet selection especially in arid and semi-arid ecosystems, which make these cattle a feasible alternative for a sustainable beef cattle production system [5]. RC cattle display remarkable foraging behavior patterns, and, consequently, a capacity to diversify their diet, especially when forages are dormant or scarce [6,7,8].

The fermentation pathways in the rumen are determined by the composition of a complex microbiome ecosystem where the host diet is the most important factor shaping it [9,10]. A greater understanding of the interactions between microorganisms and their hosting heritage cattle could help identify microorganisms with biotechnological potential, providing the potential to manipulate the microbiome or direct genetic selection to reach sustainability in extensive beef production systems [11].

In this regard, we hypothesize that the foraging behaviors and diet diversification of RC cattle in two seasons of the year impact the ruminal microbiome of these cattle differently compared to beef-specialized breeds. Although there is information about the impact of the host diet upon ruminal microbial populations and more recent studies suggest that hostile environmental conditions promoted the coevolution of such microorganisms and the host genome, little is known about the ruminal microbiome composition of locally adapted breeds grazing in harsh climate conditions [12]. The aim of this study was to characterize and compare the ruminal microbiome of three different cattle lineages (Raramuri Criollo cattle, European breeds, and a crossbreed between them) grazing on the same paddock in Northern Mexico in two different seasons.

## 2. Materials and Methods

### 2.1. Study Site

The field work was carried out at Rancho Teseachic of the Universidad Autónoma de Chihuahua (28°48′ N; 107°25′ W), located northeast of Sierra Madre Occidental, Chihuahua, Mexico. The experiment took place at a site of the ranch with a surface of 200 ha, an elevation between 2090 and 2256 AMSL, and a climate characterized by cold winters with a minimum temperature of −14 °C and hot summers with a maximum temperature of 36 °C in December and June, respectively. The average annual relative humidity is 60%, with an average annual rainfall of 580 mm characterized by one rain season per year, from June to October. Vegetation types include pine forest, oak forest, oak forest with grassland, open grassland, and shrublands [13].

### 2.2. Animals and Ruminal Liquid Sampling

All experiments and procedures were performed following approved protocols by the current Bioethics and Animal Welfare Committee of the Universidad Autónoma de Chihuahua, according to the Official Mexican Norm (NOM-062-ZOO-1999) [14].

A total of 63 cows in their second productive cycle were divided for dry (November–December) and rain (August–September) experimental seasons. The animals used represented any of three lineages shown in Table 1. Cows were kept for 45 days at the experimental location, grazing without supplementation. Twenty-four hours before taking the ruminal samples, the animals were taken to a management area of the ranch with no access to feed and free access to water. Ororuminal probes were used—one for each animal—for ruminal fluid extraction from each cow. The initial 100 mL was discarded to avoid saliva contamination and 40 mL samples were collected in conical tubes. Immediately after collection, the samples were frozen at −196 °C in liquid nitrogen and transported to the Laboratory of Biotechnology for Animal Nutrition of the Universidad Autónoma de Chihuahua.

### 2.3. DNA Extraction and 16s rRNA Gene Sequencing

A total of 15 mL of each ruminal fluid sample was filtered through a sterile gauze and centrifuged at 10,000× *g* for 10 min at 4 °C (5810R; Eppendorf, Hamburg, Germany). DNA was extracted from the resulting pellet using the ZymoBIOMICS™ DNA Miniprep Kit according to the manufacturer’s instructions (Zymo Research, Irvine, CA, USA). DNA concentration at 260 nm and the ratio 260/280 nm for purity were measured in a Nanodrop (Thermo Fisher Scientific, Walmington, DE, USA). DNA integrity was verified by electrophoresis on a 0.8% (*w*/*v*) agarose gel. The DNA samples were diluted in nuclease-free water to 10 ng/µL and the dry season samples were shipped to the MR DNA Lab (Shallowater, TX, USA), whereas the rain season samples were sent to the Metagenomics Laboratory of CICESE (Ensenada, BC, México). Further processing of samples for MiSeq sequencing on the Illumina platform were performed according to the protocols of each sequencing facility. Briefly, the V4 and V3–V4 variable regions of the 16s rRNA gene were amplified for the dry and rain seasons, respectively, and the Illumina DNA libraries were prepared. The 250 bp paired-end sequencing reactions were performed on a MiSeq platform (Illumina, San Diego, CA, USA) following the manufacturer’s instructions.

### 2.4. Bioinformatics Analyses

The output fastq files were demultiplexed using the Quantitative Insights into Microbial Ecology 2.0 (QIIME2) software package (version 2019.1) [15]. Demultiplexed sequences were filtered by a quality score (Q > 25), overlapped, and assembled into operational taxonomic units (OTUs) using the DADA2 plugin [16]. The sequence variants resulting from the DADA2 procedure, previously trimmed to retain only the V4 region by the 515F/806R primer pair for the dry season amplicons, and the V3–V4 region by the 341F/806R primer pair for rain season amplicons [17], were taxonomically classified using a naïve Bayesian classifier via the “qiime feature-classifier” command [18] with the “classify-sklearn” option against the SILVA 16s rRNA gene reference database (version 132).

The alpha diversity indices including number of observed OTUs, Shannon and Simpson indices, as well as Good’s coverage were calculated using the “qiime2-q2-diversity” command in QIIME2. Beta diversity differences between lineages were assessed with unweighted UniFrac and Bray–Curtis dissimilarity and visualized through principal coordinates analysis (PCoA) plots using QIIME2 [19,20]. The metabolic pathways of ruminal microbiome samples based on the Kyoto Encyclopedia of Genes and Genomes (KEGG) were predicted using the CowPi tool (v1.0) Galaxy Workflow [21].

### 2.5. Statistical Analysis

A two-way ANOVA was used to analyze alpha diversity indices, using lineage and season as factors. Also, a one-way ANOVA was used to compare lineages within each season in RStudio (version 1.2.1335) and the statistical package (version 3.6.0). Beta diversity analysis was carried out for unweighted UniFrac and Bray–Curtis dissimilarity by Permutational Multivariate Analysis of Variance (PERMANOVA) with 999 permutations to test differences among lineages within each season, using QIIME2 scripts [15]. Differences between lineages within each season at the taxonomic levels phylum and genus (with at least 1% of total abundance) were analyzed by a Kruskal–Wallis test, followed by a Wilcoxon test. *p*-values were adjusted for multiple comparisons using the Benjamini–Hochberg correction method. To determine significantly different taxa among lineages, the non-strict version of linear discriminant analysis (LDA) effect size (LEfSe) with a LDA score higher than 2 was performed online (https://huttenhower.sph.harvard.edu/galaxy, accessed on 5 November 2020) [22]. A heatmap in RStudio was generated to visualize the resulting taxa groups for each season. Finally, differences in the metabolic pathways across ruminal microbiome lineages, recognized by the CowPi tool (v1.0) (with >0.5% of total abundance), were identified by a Kruskal–Wallis test, followed by a Wilcoxon test. Significance was determined at *p* < 0.05.

## 3. Results

### 3.1. Alpha and Beta Diversity

A total of 1,010,882 and 1,161,295 reads (accession No: PRJNA1149031) with a mean of 36,102 and 33,180 per sample for the dry and rainy season, respectively, passed the quality control and chimera removal from Illumina paired-end raw sequences. The sequences had a mean length of 291.7 ± 10.34 and 437.98 ± 10.68 nt corresponding to the V4 and V3-V4 hypervariable regions of the 16s rRNA gene, respectively, and the Good’s coverage values were above 0.999 for all samples. The total operational taxonomic units (OTUs) obtained in the project are provided in Supplementary Files S1 and S2 for the dry and rainy seasons, respectively. The taxonomy diversity showed a total of 10,732 and 22,870 OTUs with a mean of 817.82 ± 107.74 and 983.8 ± 96.66 per sample for the dry and rainy season, respectively, with statistical difference (*p* < 0.05) between seasons and lineages (Table 2). Similarly, Simpson’s index showed significant differences between seasons and lineages whereas Shannon’s index just showed differences within seasons. On the other hand, the lineages within seasons just showed a tendency (*p* = 0.063) in the OTUs observed number between Raramuri Criollo (RC) and Crossbreed (E × RC) cattle in the rainy season (Table 2).

**Figure 1:**
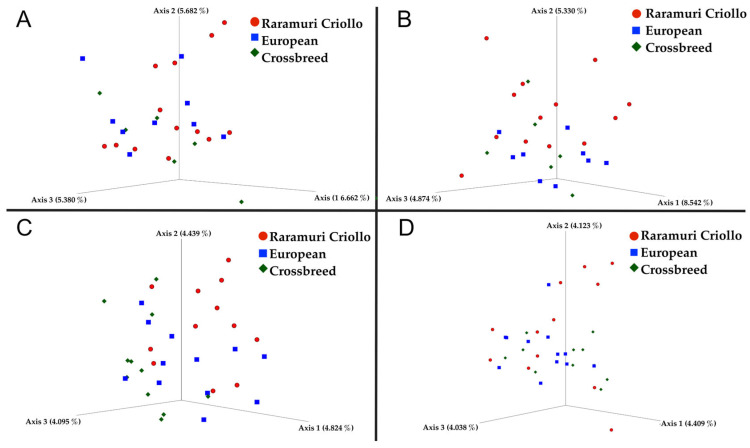
Principal coordinates analysis (PCoA) showing the different localization of bovine rumen microorganisms of three lineages. Based on the unweighted unifrac distance ((**A**,**C**) for the dry and rain seasons, respectively) and Bray–Curtis dissimilarity matrix ((**B**,**D**) for the dry and rain seasons, respectively).

Differences in beta diversity (Table 3) were found among lineages in both seasons. There was also a significant difference between the RC and the European (E) lineages for both seasons in unweighted unifrac (*p* < 0.05), as well as in Bray–Curtis for the rainy (*p* < 0.05) and dry (*p* = 0.05) seasons. There was only a tendency (*p* < 0.10) towards a difference in beta diversity between RC and E × RC cattle in the rainy season with both methods, and no differences (*p* > 0.10) between E × RC and E groups. The bacterial communities were separated from one another in lineages within seasons through PCoA, based on the unweighted unifrac matrix and Bray–Curtis dissimilarity matrix for the dry and rainy seasons (Figure 1).

### 3.2. Relative Prokaryotic Abundance

A total of 21 and 19 bacterial phyla were identified across the samples for the dry (Table 4) and rainy (Table 5) seasons, respectively. The most abundant phyla, representing above 1% of the total bacterial and archaeal community in the dry season were: Bacteroidetes, Firmicutes, Euryarchaeota, Patescibacteria, and Proteobacteria, without differences (*p* > 0.05) between lineages (Table 4). Meanwhile, in the rainy season (Table 5) the most abundant phyla were: Bacteroidetes, Firmicutes, Planctomycetes, and Proteobacteria, in which no differences (*p* > 0.05) between lineages were found, and Patescibacteria and Spirochaetes, for which statistical differences (*p* < 0.05) were found between the RC and E lineages, with intermediate values for these phyla in E × RC. However, when the raw *p*-values were adjusted for false discovery rate (FDR) this significance was lost (*p* = 0.14). In the comparison of the most abundant phyla between the dry and rainy seasons, Planctomycetes and Spirochaetes represented above 1% of the microbial community in the rainy season but below 1% in the dry season, whereas the phylum Euryarchaeota represented around 3% of the community in the dry season and below 1% in the rainy season.

The bacterial community with intermediate abundance in the dry (Table 4) and rain (Table 5) seasons represented between 0.1 to 1% and did not show differences (*p* > 0.05) between lineages. The less abundant phyla represented below 0.1% of the total abundance. In the dry season after adjusting for FDR, the phylum Armatimonadetes was higher (*p* = 0.01) in the E group compared to the RC and E × RC lineages, whereas for the rainy season, the abundance of the phylum Epsilonbacteraeota tended to be different (*p* = 0.06) between the RC and E groups, with intermediate values in the E × RC cross. In these less abundant microbial groups, the phyla Armatimonadetes and Fusobacteria were detectable in the dry season but not in the rainy season.

### 3.3. Differences in Bacterial Groups

**Figure 2:**
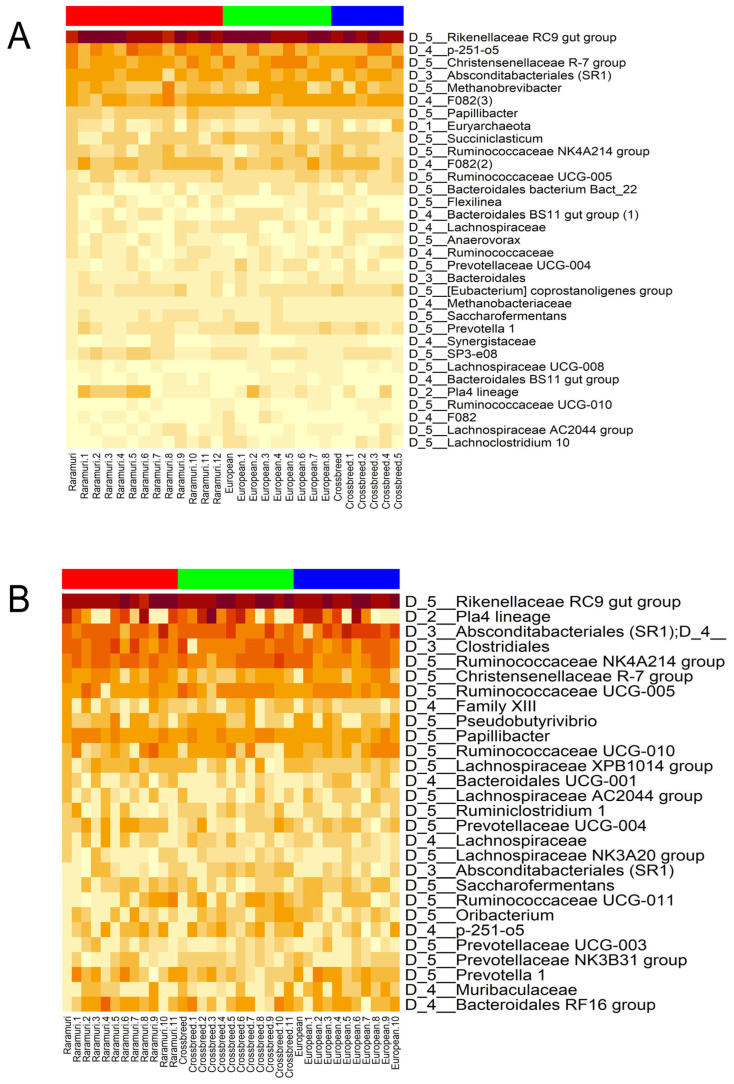
Heatmap of ruminal core microbiomes of three cattle lineages (Raramuri Criollo = Red, European = Green; E × RC = Blue) in the dry (**A**) and rainy (**B**) seasons. The reported microbial groups were identified in at least 50% of samples. The number provided before the microbial taxa represents the taxonomic level (D_1: Phylum; D_2: Class; D_3: Order; D_4: Family; D_5: Genus).

A total of 33 and 28 microbial groups for the dry (Figure 2A) and rainy (Figure 2B) season, respectively, were identified in at least 50% of the total samples, varying their abundances per season. The microbial groups include mainly genera; however, there are phyla, class, orders, and families as shown in Figure 2A,B.

**Figure 3:**
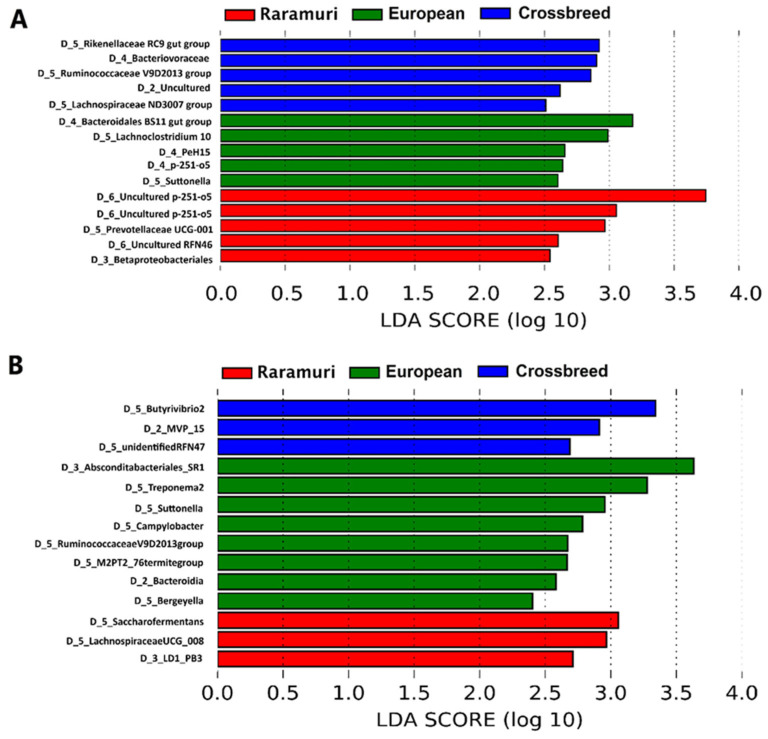
Linear discriminant analysis effect size (LEfSe) to determine biomarker microorganisms within the rumen microbial community of different lineages for the dry (**A**) and the rainy (**B**) seasons. The taxonomic levels of the microbial groups are indicated by the prefixes: D_2: Phylum; D_3: Class; D_4: Order; D_5: Family; D_6: Genus.

To identify key microbial groups that explain the differences between lineages, a LEfSe analysis on its non-strict version was used. Biomarker microorganisms with an LDA score of at least 2 were identified as different among groups. A total of 15 bacterial groups (five for each lineage) were recognized differentially across the lineages in the dry season (Figure 3A), while in the rainy season (Figure 3B) 14 bacterial groups were identified (3, 8, and 3 bacterial groups for RC, E and E × RC, respectively).

Dominant groups of microorganisms that are represented in more than 1% of the total abundance at the genus level are shown in Table 6 and Table 7 for the dry and rainy seasons, respectively. After adjusting the raw *p*-values for FDR non-significant statistical differences were found between lineages.

### 3.4. KEGG-Predicted Ruminal Microbiome Pathways in the Dry and the Rainy Seasons

**Figure 4:**
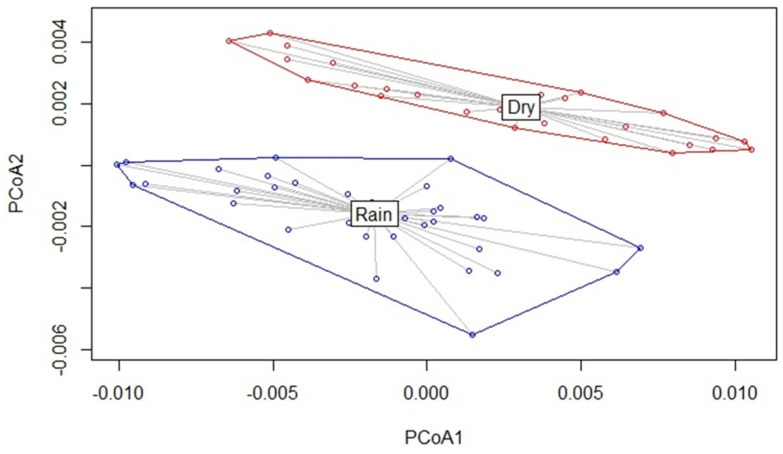
Principal coordinate analysis (PCoA) showing the different localization of seasons based on Bray–Curtis distances of predicted metabolic pathways in ruminal microorganisms, identified by the CowPi tool.

**Figure 5:**
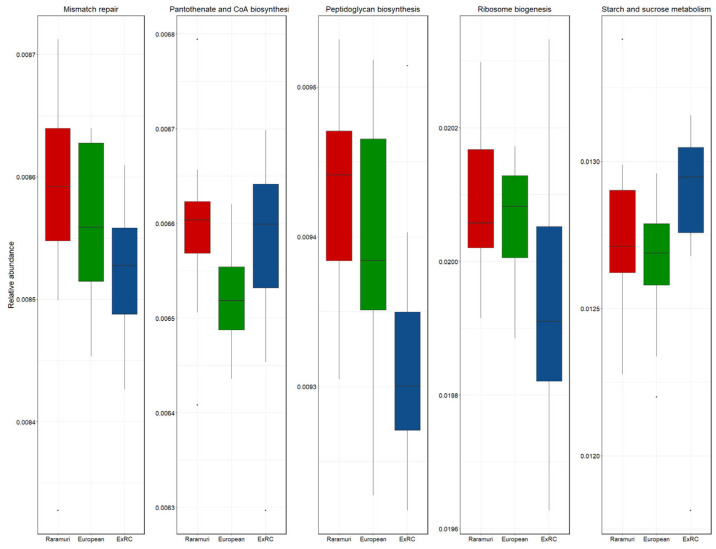
Boxplot of mismatch repair, pantothenate, CoA biosynthesis, peptidoglycan biosynthesis, ribosome biogenesis, and starch and sucrose metabolism predicted pathways for bacteria isolated from the rumen of different lineage cows during the rainy season.

The main KEGG-predicted pathways (with at least 0.5% of total abundance) identified by the CowPi tool include the same 64 metabolic pathways for both the dry and rainy seasons; the complete lists are provided in Supplementary Files S4 and S5. The differences in functional metabolic pathways by season are shown in the PCoA analysis (Figure 4). Predicted mismatch repair pathways were higher (*p* < 0.05) in the E than in the E × RC lineage but not greater than in the RC lineage in the dry season (Figure S1). For the rainy season (Figure 5), RC and E × RC differed (*p* < 0.05) in predicted peptidoglycan biosynthesis pathways and there was a tendency (*p* < 0.10) towards mismatch repair and ribosome biogenesis between the RC and E × RC groups, while for predicted pantothenate and CoA biosynthesis and starch and sucrose metabolism only subtle differences were found, with tendencies between RC vs. E and E × RC vs. E, respectively.

## 4. Discussion

The importance of the utilization of Raramuri Criollo cattle (RC) as a strategy to face the increasingly exacerbated challenges of climate change in the beef cattle production system is clear, especially in arid and semi-arid ecosystems [5]. However, increasing knowledge about this type of livestock is necessary for the development of new biotechnologies and to accomplish a balance between these, genetic selection, and protection of the environment. The microbiome characterization of RC cattle carried out in this work provides interesting data on the rusticity of these animals and their adaptation to harsh conditions.

The OTUs obtained by sequencing the V4 and V3–V4 hypervariable regions of the 16s rRNA gene were similar to the 12,361 and 14,561 reported in other studies on beef and dairy cattle, respectively [23,24]. Microbial diversities were analyzed using Shannon’s and Simpson’s indices, given their suitability for this kind of data. The results for alpha diversity were similar to those reported by [25,26] in both indexes, and are considered to be good for the highly complex ecosystems found in the rumen. The statistical differences in Shannon’s and Simpson’s indexes between lineages and seasons could be due to the sequenced region. The evaluation of the impact of sequencing either the V3 or the V3–V4 region in microbiome studies has shown that the number of amplicon sequence variants (ASV) is higher when sequencing the V3-V4 region. Thus, it is recommended to report ASVs for V3 and from V3–V4 regions separately [27]. Following the same criteria, we independently analyzed and reported OTUs from the V3 and V3–V4 reads.

On the other hand, the results obtained for beta diversity indexes showed differences in the microbial populations between RC and European (E) lineages, but not between the crossbred (E × RC) and pure lineages (Table 3). This has also been reported for beef cattle breeds, where Bray–Curtis dissimilarity was different between pure Angus and pure Charolais animals, but no differences were found with the crossbred [28,29]. In our study, the lineage effect on beta diversity was different in each season (Table 3). Seasonal precipitation events increase the water availability and modify the dynamics of the plant species in the ecosystem [30], with a greater variety of vegetation available during the rainy season, which, combined with the particular foraging behavior of RC cattle [13,31], explains the differences in microbiome diversity between linages.

The phyla Bacteroidetes, Firmicutes, Patescibacteria, and Proteobacteria were found as dominant in the three lineages for both seasons, similar to the findings in other microbiome studies in Nellore, Angus, and Limousin beef cattle breeds [24,32] and in grazing dairy cattle [33].

Regarding the less abundant phyla, Armatimonadetes in the dry season and Epsilonbacteraeota in the rainy season had higher relative abundances in E cattle (Table 4 and Table 5). The effect of Armatimonadetes in the rumen environment is still unknown, since this phylum was recently classified and its species started to be identified only in the last decade [34,35]. While, Epsilonbacteraeota has been negatively correlated with dry matter intake and positively correlated with acetate and propionate production [36].

The groups of microorganisms with presence in at least 50% of the samples (Figure 2) as well as the microorganisms at the genus level represented in more than 1% of the total abundance (Table 6 and Table 7), reflect the presence of a “core microbiome” across all animals, reinforcing the existence of such microbial community, as reported by other research groups [9,37,38]. Some microorganisms within this core have similar functions. Such a functional redundancy demonstrates the importance of maintaining the essential physiological processes regardless of the phylogenetic variations caused by minor groups [39]. Several members of this core microbiome, such as *Rikenellaceae* RC9, *Ruminococcaceae*, *SP3* e08, *Eubacterium*, *U-Lachnospiraceae*, and *Ruminococcus* 1 have fibrolytic functions involved in the degradation of cellulose, hemicellulose, and xylan [38,40,41,42,43]. On the other hand, *Prevotellaceae* participates in protein catabolism, whereas *Bacteroidales* and *Prevotella* are overlapping in polysaccharide and protein degradation [44]. *Bacteroidales* is considered a “keystone group” for its pivotal role in the ruminal microbiome of grazing yak, which live in harsh environmental conditions and, similar to RC cattle, have adaptative characteristics such as the capacity to diversify its diet with woody plants [45]. Furthermore, some genera such as *Christensenellaceae*, *U-Muribaculaceae*, *Succiniclasticum*, *Papillibacter,* and *Saccharofermentans* produce volatile fatty acids as acetic, butyric, and propionic [46,47,48,49,50], whereas *Methanobrevibacter* is involved in methane production [51]. Finally, *Anaerovorax* participates in ruminal biohydrogenation [52]. The function of *U-Absconditabacteriales* in rumen is unknown since it was recently identified [53].

The LEfSe analysis identified key bacterial groups for the cattle lineages in each season of this study. The presence of *Uncultured p-251-o5* and *Uncultured RFN46* in the ruminal microbiome of RC cattle in the dry season reflect its singularity, because although their function in the microbial ecosystem is unknown, their presence could be associated with characteristics of rusticity reported in animals adapted to harsh environmental conditions, including yak, buffalo, and locally adapted cattle breeds, such as Maremmana and Aubrac [36,54,55]. Moreover, the *Uncultured p-251-o5* is a key species in grazing yak, due to its interaction with several other groups in the ruminal ecosystem [45]. The identification of fibrolytic enzymes producers such as *Prevotellaceae UCG-001* [56,57] in RC cattle point towards the contribution of this genus to the capacity that RC has to include in its diet plants with more fibrous compounds [58]. The *Prevotellaceae UCG-001* group is positively correlated with metabolic pathways including AMPK signaling, sphingolipid signaling, and dopaminergic synapse, and negatively correlated with arachidonic acid metabolism and thyroid hormone synthesis [59]. The AMPK signaling pathway is one of the mechanisms that protects the rumen from the inflammation caused by heat stress [60]; thus, the presence of *Prevotellaceae UCG-001* would add to the results by [31], who reported that Mexican RC cattle have a greater thermotolerance than European breeds in the arid conditions of the Chihuahuan desert.

On the other hand, the sphingolipid signaling pathway is involved in both apoptosis and proliferation processes in eukaryotic cells and has been reported to occur in ruminal epithelial cells of goats [61]. These cellular processes could have an impact upon the functionality of the ruminal absorption surface, providing a defense against pathogen organisms and making the absorption of nutrients more efficient [62]. Regarding the dopaminergic synapsis pathway, to which *Prevotellaceae UCG-001* is positively correlated, it influences locomotor activity, memory, learning, and endocrine regulation in mammals [63]. These physiological processes have an impact on grazing patterns in ruminants and stimulates search and consumption behaviors [64]. There was a tendency of RC to stay grazing for longer periods of time [31]; therefore, it would be interesting to explore these metabolic pathways in RC cattle, related to their grazing behavior.

Additionally, the microbial groups identified by the LEfSe analysis for the rainy season also reflect particular characteristics of adapted ruminants living in rough environmental conditions. The genus *Lachnospiraceae UCG-008* has been reported as a differential group in yaks grazing on high shrub-coverage areas compared to yaks grazing on minor shrub-coverage areas [65]. Similarly, the presence of *Lachnospiraceae UCG-008* in RC could contribute to the capacity of these cattle to diversify their diet and including the more abundant number of plants available in the rainy season. Moreover, in our study the *LD1-PB3* order shows up as a biomarker for RC cattle. *LD1-PB3* belongs to the *Verrucomicrobia* phylum, a differential microbial group in the rumen of healthy goats [61]. Also coming up as a RC biomarker microorganism is the *Saccharofermentans* genus, which has been associated with improved milk fat and other functional components of milk in Holstein cows [66]; these beneficial effects could extend to RC in extensive environmental conditions.

The microorganisms identified by the LEfSe analysis for the E lineage in the dry season include the *Bacteriodales BS11* gut group and the genus *Lachnoclostridium 10*, which are characterized by their activity in non-specific carbohydrate degradation, including starch and fiber, and are also capable of producing VFAs [54,67,68]. *Sutonella* is a gram-negative coccobacillus implicated in fiber, glucose, and sucrose degradation and characterized by indole production, a metabolite involved in the cellular signaling of pathogen microorganisms such as *Escherichia coli*, *Edwardsiella tarda,* and *Vibrio cholerae* [69,70]. Since the *PeH15* family was only recently reported in the rumen ecosystem, its functions are still unknown [71]. For the rainy season, the biomarker microorganisms for the European lineage included groups as *Absconditabacteriales SR1*, which has been reported in the ruminal microbiome of dairy cows with deteriorated health [72]. Furthermore, the *Treponema*, *Campylobacter*, and *Ruminococcaceae* groups have been associated with carbohydrate degradation in ruminal ecosystems [73,74,75], and the *M2PT2-76 termite* group and *Bergeyella* have been reported in Holstein and Belgian blue cattle, respectively, although their ruminal functions are still unclear [76].

Finally, the E × RC group shows differentiated microorganisms identified by LEfSe in the dry season with similar functions to those discussed for the two pure breeds. The families *Rikenellaceae RC9 gut group* and *Ruminococcaceae V9D2013* are involved in fiber and starch metabolization, propionic acid production, and biohydrogenation [36,54,77,78,79,80]. Similarly, *Lachnospiraceae ND3007* is a cellulolytic microorganism, widely reported in ruminal ecosystems [81,82]. The ruminal function of *Bacteriovoraceae* is still unknown, although this family is part of the *Deltaproteobacteria* class, which is characterized by the sulfate reduction to sulfide that eventually could be used as amino acid precursor for another ruminal microorganisms [83]. For the rainy season, the differences in the microbial biomarkers for the crossbreed lineage compared to the pure breed lineages included *Butyrivibrio* which is involved in ruminal biohydrogenation [84] and *MVP-15* that has been reported in Hereford x Angus cross but its ruminal function is also unknown [85].

The CowPi results based on KEGG show the general functions of microbial ecosystems, including pathways that are part of the cellular metabolism, genetic information processing, and environmental information processing, which are consistent with previous ruminal microbiome studies [86,87]. Even though in this study different, overlapping regions were sequenced for each season, the difference observed in Figure 4 aligns with findings from other research groups, which have reported a clear distinction in the ruminal bacterial community structure influenced by climate and diet [9]. The only statistically different (*p* < 0.05) predicted pathway between the E and E × RC lineages in the dry season was the mismatch repair system, which was also different in the rainy season. This pathway is related to genomic stability and has been reported in ruminal ecosystems [88]. Similarly, predicted peptidoglycan biosynthesis is the only pathway statistically different (*p* < 0.05) between RC and E × RC lineages in the rainy season and is higher in animals with an efficient feed conversion [89]. Additionally, the predicted microbial pantothenate and CoA biosynthesis pathways only showed a difference tendency (*p* < 0.10) between RC and E lineages in this study. Metabolites of these pathways are found at a higher serum concentration in feed-efficient Angus steers compared to the low-efficient ones, thus playing a role in genome–microbiome interactions [90]. This pathway occurs in the rumen microbiome of yaks [91] and could be related to cellulolytic bacteria in the ruminal ecosystem [92]. The absence of other statistical differences in the analyses of functional microbiome pathways could be due to software limitations of the PICRUSt (v.1.1.4) and CowPi (v1.0) tools at the time of this study, as well as to the yet unclassified bacteria and their underestimated microbial pathways and functions [21].

## 5. Conclusions

The function of biomarker microorganisms identified in this study suggest the ruminal adaptation of Raramuri Criollo cattle to the harsh environmental conditions of northern Mexico, since these microbial groups are specialized in the degradation of cacti, forbs, and shrub compounds, and have been identified in other cattle breeds and adapted ruminant species. Thus, the groups *U-Bacteroidales 3*, *Uncultured p-251-o5*, *Prevotellaceae UCG-001*, *Lachnospiraceae UCG-008*, and *Verrucomicrobia* carry out functions related to the utilization of resources and adaptation to specially challenging environmental conditions.

These results add to the identification of heritage cattle as a relevant resource in the current scenario of global climate change. Future studies on the advantageous features that might have resulted in the rumen of Raramuri Criollo cattle could shed light into the generation of technologies for accurate animal selection and for manipulation of ruminal microbiota. Further research should also include the identification of eukaryotic microorganisms, the expression of key microbial genes for nutrient degradation, as well as the expression in rumen epithelial cells genes that differentially impact the absorption of nutrients in the Raramuri Criollo compared to European lineages.

## Figures and Tables

**Table 1:** Number of animals and lineages used in each experimental season.

Season	Animals		
	RC	E	E × RC
Rain	12	11	12
Dry	13	9	6

RC: Raramuri Criollo purebred, E: European (Angus and Hereford), E × RC: European × Raramuri Criollo.

**Table 2:** Alpha diversity indices in the ruminal microbiome among season, cow lineage, and lineage within each season.

Comparison		Index					
		Observed OTUs		Simpson’s Index		Shannon’s Index	
		Mean ± SE	*p*-Value	Mean ± SE	*p*-Value	Mean ± SE	*p*-Value
Lineage	RC	865.6 ± 21.8 ^a^	0.015	0.996 ± 0.0002 ^a^	0.045	9.01 ± 0.04	0.169
	E	924.3 ± 28.9 ^b^		0.996 ± 0.0003 ^a^		9.05 ± 0.05	
	E × RC	955.9 ± 34.6 ^b^		0.997 ± 0.0002 ^b^		9.11 ± 0.04	
Season	Dry	817.8 ± 20.4 ^a^	>10^−4^	0.995 ± 0.0001 ^a^	>10^−4^	8.93 ± 0.03 ^a^	>10^−4^
	Rain	983.8 ± 16.3 ^b^		0.997 ± 0.0001 ^b^		9.15 ± 0.03 ^b^	
Lineage in dry season	RC	803.8 ± 25.4	0.816	0.995 ± 0.0002	0.347	8.93 ± 0.06	0.902
	E	833.7 ± 33.3		0.995 ± 0.0003		8.91 ± 0.06	
	E × RC	824.3 ± 65.2		0.996 ± 0.0002		8.95 ± 0.03	
Lineage in rainy season	RC	932.5 ± 24.7	0.063	0.997 ± 0.0002	0.927	9.09 ± 0.05	0.324
	E	998.4 ± 30.6		0.997 ± 0.0001		9.16 ± 0.05	
	E × RC	1021.6 ± 25.1		0.997 ± 0.0002		9.19 ± 0.04	

^a,b^ Values within comparison with different superscripts are statistically different (*p* < 0.05). RC: Raramuri Criollo purebred, E: European, E × RC: European × Raramuri Criollo.

**Table 3:** Beta diversity comparison of ruminal microbiome from cows with different lineages in two seasons based on the unweighted unifrac and Bray–Curtis PERMANOVA.

Season	Lineage	Comparison	Sample Size (Cows)	Permutations	*p*-Value	
					Unweighted Unifrac	Bray–Curtis
Dry	RC	E	22	999	0.039 *	0.052
		E × RC	19	999	0.105	0.887
	E	E × RC	15	999	0.348	0.769
Rain	RC	E	23	999	0.018 *	0.003 *
		E × RC	24	999	0.085	0.063
	E	E × RC	23	999	0.232	0.115

* Statistical difference (*p* < 0.05) between groups. RC: Raramuri Criollo, E: European, E × RC: European × Raramuri Criollo.

**Table 4:** Relative abundance of prokaryotic phyla in the rumen of cattle of different lineages in the dry season.

Phylum	Lineage			*p*-Value	^z^*p*-Value
	Raramuri Criollo	European	E × RC		
Above 1% of community					
Bacteroidetes	53.99 ± 6.15	53.47 ± 8.45	53.02 ± 8.48	0.97	0.99
Firmicutes	33.08 ± 5.09	34.09 ± 7.49	33.23 ± 6.74	0.99	0.99
Euryarchaeota	3.026 ± 1.34	2.943 ± 1.53	3.041 ± 1.25	0.89	0.99
Patescibacteria	2.123 ± 0.53	2.170 ± 0.46	2.212 ± 0.77	0.99	0.99
Proteobacteria	1.195 ± 0.40	1.047 ± 0.15	1.247 ± 0.31	0.45	0.99
Between 0.1 and 1% of community					
Kiritimatiellaeota	0.997 ± 0.39	0.866 ± 0.47	1.171 ± 0.32	0.29	0.96
Spirochaetes	0.860 ± 0.18	0.872 ± 0.52	0.774 ± 0.15	0.36	0.96
Planctomycetes	0.904 ± 0.57	0.663 ± 0.30	0.636 ± 0.38	0.66	0.96
Chloroflexi	0.776 ± 0.46	0.698 ± 0.29	0.874 ± 0.63	0.96	0.96
Tenericutes	0.636 ± 0.17	0.713 ± 0.19	0.665 ± 0.03	0.80	0.96
Actinobacteria	0.483 ± 0.27	0.506 ± 0.30	0.728 ± 0.58	0.70	0.96
Synergistetes	0.502 ± 0.17	0.472 ± 0.08	0.484 ± 0.11	0.95	0.96
Fibrobacteres	0.259 ± 0.22	0.214 ± 0.11	0.261 ± 0.19	0.81	0.96
Verrucomicrobia	0.158 ± 0.06	0.185 ± 0.07	0.290 ± 0.23	0.65	0.96
Lentisphaerae	0.126 ± 0.77	0.140 ± 0.06	0.133 ± 0.03	0.82	0.96
Elusimicrobia	0.167 ± 0.13	0.101 ± 0.07	0.093 ± 0.09	0.44	0.96
Below 0.1% of community					
WPS-2	0.068 ± 0.05	0.067 ± 0.05	0.016 ± 0.02	0.08	0.20
Cyanobacteria	0.058 ± 0.05	0.062 ± 0.03	0.128 ± 0.14	0.31	0.39
Armatimonadetes	0.026 ± 0.02 ^a^	0.062 ± 0.02 ^b^	0.015 ± 0.01 ^a^	0.002 *	0.01 *
Epsilonbacteraeota	0.011 ± 0.01	0.027 ± 0.03	0.018 ± 0.01	0.26	0.39
Fusobacteria	0.001 ± 0.00	0.002 ± 0.00	0.000 ± 0.00	0.71	0.71

* Statistical difference (*p* < 0.05) between groups. ^a,b^ Values within the same row with different superscripts are statistically different (*p* < 0.05). ^z^*p*-value is an adjusted Benjamini–Hochberg correction value.

**Table 5:** Relative abundance of prokaryotic phyla in the rumen of cattle of different lineages in the rainy season.

Phylum	Lineage			*p*-Value	^z^*p*-Value
	Raramuri Criollo	European	E × RC		
Above 1% of community					
Bacteroidetes	50.89 ± 5.55	50.04 ± 6.08	49.14± 4.97	0.84	0.84
Firmicutes	37.81 ± 5.36	35.38 ± 6.60	37.30 ± 7.05	0.56	0.67
Patescibacteria	1.96 ± 0.85 ^a^	2.93 ± 1.27 ^b^	2.25 ± 0.64 ^ab^	0.05 *	0.14
Proteobacteria	2.11± 0.66	2.69 ± 1.02	2.78 ± 2.39	0.15	0.44
Planctomycetes	1.66 ± 1.65	2.74 ± 2.47	2.55 ± 2.84	0.47	0.67
Spirochaetes	1.25 ± 0.37 ^a^	1.86 ± 0.57 ^b^	1.47 ± 0.52 ^ab^	0.04 *	0.14
Between 0.1 and 1% of community					
Elusimicrobia	0.14 ± 0.06	0.18 ± 0.11	0.13 ± 0.10	0.44	0.74
Kiritimatiellaeota	0.74 ± 0.24	0.94 ± 0.46	1.00 ± 0.45	0.28	0.74
Euryarchaeota	0.70 ± 0.47	0.51 ± 0.30	0.73 ± 0.38	0.38	0.74
Chloroflexi	0.53 ± 0.33	0.34 ± 0.13	0.40 ± 0.21	0.27	0.74
Tenericutes	0.44 ± 0.19	0.55 ± 0.28	0.46 ± 0.27	0.54	0.75
Actinobacteria	0.45 ± 0.22	0.34 ± 0.22	0.41 ± 0.31	0.61	0.75
Synergistetes	0.24 ± 0.16	0.19 ± 0.05	0.17 ± 0.06	0.68	0.75
Fibrobacteres	0.36 ± 0.37	0.44 ± 0.30	0.40 ± 0.35	0.82	0.82
Verrucomicrobia	0.24 ± 0.15	0.21 ± 0.12	0.15 ± 0.07	0.44	0.74
WPS-2	0.05 ± 0.05	0.11 ± 0.08	0.13 ± 0.10	0.06	0.59
Below 0.1% of community					
Lentisphaerae	0.05 ± 0.06	0.06 ± 0.06	0.06 ± 0.04	0.95	0.95
Cyanobacteria	0.09 ± 0.13	0.07 ± 0.06	0.11 ± 0.12	0.48	0.73
Epsilonbacteraeota	0.00 ± 0.01 ^a^	0.08 ± 0.11 ^b^	0.03 ± 0.06 ^ab^	0.02 *	0.06

* Statistical difference (*p* < 0.05) between groups. ^a,b^ Values within the same row with different superscripts are statistically different (*p* < 0.05). ^z^*p*-value is an adjusted BenjaminiHochberg correction value.

**Table 6:** Prokaryotic genera with relative abundance above 1% in the rumen of cattle from different lineages within the dry season.

Genus/ Specific OTU	Phylum	Lineage			*p*-Value	^z^*p*-Value
		Raramuri Criollo	European	E × RC		
*Rikenellaceae RC9*	*Bacteroidetes*	27.40 ± 4.58	28.47 ± 4.20	27.49 ± 3.80	0.84	0.99
*Christensenellaceae*	*Firmicutes*	5.87 ± 1.42	6.10 ± 2.85	5.97 ± 2.15	0.99	0.99
*U-Bacteroidales 1*	*Bacteroidetes*	4.60 ± 0.84	4.02 ± 0.60	4.75 ± 0.73	0.16	0.85
*Prevotella*	*Bacteroidetes*	2.92 ± 0.79	3.18 ± 0.98	3.67 ± 1.12	0.33	0.98
*U-Bacteroidales 3*	*Bacteroidetes*	2.93 ± 1.01 ^a^	1.88 ± 0.76 ^b^	2.34 ± 1.06 ^ab^	0.04 *	0.43
*U-Bacteroidales 2*	*Bacteroidetes*	2.88 ± 0.65	2.62 ± 0.64	2.75 ± 0.56	0.57	0.99
*Ruminococcaceae NK4A214*	*Firmicutes*	2.11 ± 0.59	2.14 ± 0.80	2.09 ± 0.81	0.84	0.99
*U-Absconditabacteriales*	*Patescibacteria*	1.94 ± 0.49	2.02 ± 0.37	2.07 ± 0.70	0.99	0.99
*Methanobrevibacter*	*Euryarchaeota*	1.73 ± 0.95	1.82 ± 1.09	1.78 ± 0.75	0.81	0.99
*SP3 e08*	*Bacteroidetes*	1.70 ± 0.32	1.78 ± 0.35	2.06 ± 0.38	0.20	0.85
*Ruminococcaceae UCG-010*	*Firmicutes*	1.61 ± 0.19	1.56 ± 0.17	1.51 ± 0.45	0.50	0.99
*Eubacterium*	*Firmicutes*	1.55 ± 0.37	1.71 ± 0.56	1.57 ± 0.59	0.75	0.99
*U-Muribaculaceae*	*Bacteroidetes*	1.28 ± 0.38	1.52 ± 0.38	1.25 ± 0.27	0.27	0.96
*Prevotellaceae UCG 003*	*Bacteroidetes*	1.26 ± 0.40	1.24 ± 0.32	1.18 ± 0.60	0.89	0.99
*Succiniclasticum*	*Firmicutes*	1.18 ± 0.29	1.39 ± 0.23	1.34 ± 0.40	0.16	0.85
*U-Lachnospiraceae*	*Firmicutes*	1.16 ± 0.33	1.16 ± 0.52	1.11 ± 0.44	0.96	0.99
*Ruminococcaceae UCG-014*	*Firmicutes*	1.10 ± 0.34	0.96 ± 0.25	0.96 ± 0.32	0.42	0.99
*Anaerovorax*	*Firmicutes*	1.07 ± 0.26	1.05 ± 0.23	1.08 ± 0.28	0.85	0.99
*Papillibacter*	*Firmicutes*	1.04 ± 0.20	0.98 ± 0.23	1.05 ± 0.25	0.90	0.99
*Ruminococcus 1*	*Firmicutes*	0.92 ± 0.28 ^a^	1.32 ± 0.29 ^b^	0.99 ± 0.38 ^ab^	0.04 *	0.43
*Saccharofermentans*	*Firmicutes*	1.02 ± 0.18	1.22 ± 0.37	1.12 ± 0.29	0.43	0.99

* Statistical difference (*p* < 0.05) between groups. ^a,b^ Values within the same row with different superscripts are statistically different (*p* < 0.05). ^z^*p*-value is an adjusted Benjamini–Hochberg correction value.

**Table 7:** Prokaryotic genera with relative abundance above 1% in the rumen of cattle from different lineages within the rainy season.

Genus/ Specific OTU	Phylum	Lineage			*p*-Value	^z^*p*-Value
		Raramuri Criollo	European	E × RC		
*Rikenellaceae RC9*	*Bacteroidetes*	19.03 ± 2.31	19.66 ± 3.02	19.58 ± 3.33	0.66	0.78
*Prevotella*	*Bacteroidetes*	8.13 ± 3.82	7.41 ± 1.67	8.03 ± 1.49	0.61	0.78
*F082*	*Bacteroidetes*	7.11 ± 1.76	5.87 ± 0.80	5.93 ± 0.89	0.06	0.62
*Christensenellaceae*	*Firmicutes*	5.45 ± 1.17	4.45 ± 1.33	4.70 ± 1.53	0.22	0.62
*Ruminococcaceae UCG-010*	*Firmicutes*	2.54 ± 0.56	3.23 ± 1.50	2.75 ± 1.15	0.68	0.78
*Ruminococcaceae NK4A214*	*Firmicutes*	2.53 ± 0.74	2.34 ± 0.50	2.55 ± 0.74	0.71	0.78
*Muribaculaceae*	*Bacteroidetes*	2.54 ± 1.53	2.46 ± 0.94	1.77 ± 0.86	0.40	0.78
*Prevotellaceae UCG 003*	*Bacteroidetes*	2.14 ± 0.59	2.30 ± 0.65	2.10 ± 0.63	0.78	0.78
*Prevotellaceae UCG 001*	*Bacteroidetes*	1.56 ± 0.49	1.90 ± 0.64	1.50 ± 0.78	0.21	0.62
*p-251-o5*	*Bacteroidetes*	1.45 ± 1.07	1.79 ± 0.89	1.84 ± 0.82	0.23	0.62
*Ruminococcaceae UCG-005*	*Firmicutes*	1.27 ± 0.48	1.48 ± 0.25	1.38 ± 0.29	0.24	0.62
*Family XIII AD3011*	*Firmicutes*	1.27 ± 0.66	0.99 ± 0.50	1.17 ± 0.34	0.57	0.78
*Succiniclasticum*	*Firmicutes*	1.10 ± 0.55	1.22 ± 0.41	1.21 ± 0.43	0.74	0.78

^z^*p*-value is an adjusted Benjamini–Hochberg correction value.

## Data Availability

The metagenome reads are available in the NCBI database under the accession number PRJNA1149031.

## Supplementary Materials

The following supporting information can be downloaded at: https://www.mdpi.com/article/10.3390/microorganisms12112203/s1, File S1: Total OTUs dry season; File S2: Total OTUs rainy season; File S3: KEGG predicted pathways of ruminal microbiome in the dry season; File S4: KEGG predicted pathways of ruminal microbiome in the dry season; Figure S1: Boxplot of predicted mismatch repair pathway for bacteria isolated from the rumen of different lineage cows during the dry season.
