# Supplementary material for: The Rumen Microbiome Composition of Raramuri Criollo and European Cattle in an Extensive System
Source: Microorganisms. 2024 Oct 31;12(11):2203. doi: 10.3390/microorganisms12112203 (PMC11596369; doi:10.3390/microorganisms12112203)
Supplement: Supplementary file 1 [file microorganisms-12-02203-s001.zip › Figure S1.pdf]

Supplementary Material

The Rumen Microbiome Composition of Raramuri Criollo and European Cattle in an Extensive System

Adrian Maynez-Perez [1], Francisco J. Jahuey-Martínez [1], José A. Martínez-Quintana [1], Michael E. Hume [2], Robin C. Anderson [2], Agustín Corral-Luna [1], Felipe A. Rodríguez-Almeida [1], Yamicela Castillo-Castillo [1] and Monserrath Felix-Portillo [1,*]

[1] Facultad de Zootecnia y Ecología, Universidad Autónoma de Chihuahua, Chihuahua 31453, Chih., Mexico; amaynez@uach.mx (A.M.-P.); fjahuey@uach.mx (F.J.J.-M.); jomartinez@uach.mx (J.A.M.-Q.); acorral@uach.mx (A.C.-L.); frodrigu@uach.mx (F.A.R.-A.); ycastillo@uach.mx (Y.C.-C.)

[2] Food and Feed Safety Research Unit, Southern Plains Area Research Center, United States Department of Agriculture, Agricultural Research Service, College Station, TX 77845, USA; mehume@suddenlink.net (M.E.H.); robin.anderson@usda.gov (R.C.A.)

* Correspondence: monserrath.felix@uach.mx

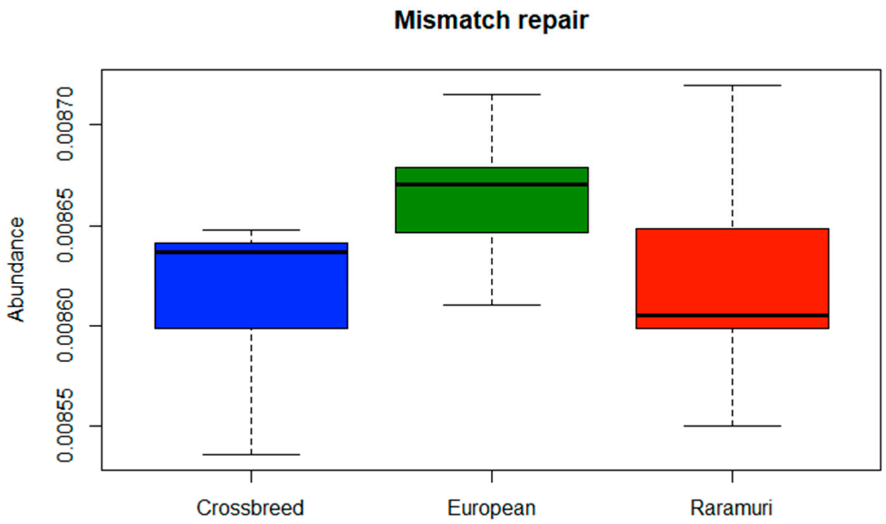

Figure S1. Boxplot of predicted mismatch repair pathway for bacteria isolated from the rumen of different lineage cows during the dry season.
